# Variety Identification of Raisins Using Near-Infrared Hyperspectral Imaging

**DOI:** 10.3390/molecules23112907

**Published:** 2018-11-08

**Authors:** Lei Feng, Susu Zhu, Chu Zhang, Yidan Bao, Pan Gao, Yong He

**Affiliations:** 1 College of Biosystems Engineering and Food Science, Zhejiang University, Hangzhou 310058, China; lfeng@zju.edu.cn (L.F.); sszhu@zju.edu.cn (S.Z.); chuzh@zju.edu.cn (C.Z.); ydbao@zju.edu.cn (Y.B.); 2 Key Laboratory of Spectroscopy Sensing, Ministry of Agriculture and Rural Affairs, Hangzhou 310058, China; 3 College of Information Science and Technology, Shihezi University, Shihezi 832000, China; 4 State Key Laboratory of Modern Optical Instrumentation, Zhejiang University, Hangzhou 310058, China

**Keywords:** near-infrared hyperspectral imaging, raisins, support vector machine, pixel-wise, object-wise

## Abstract

Different varieties of raisins have different nutritional properties and vary in commercial value. An identification method of raisin varieties using hyperspectral imaging was explored. Hyperspectral images of two different varieties of raisins (Wuhebai and Xiangfei) at spectral range of 874–1734 nm were acquired, and each variety contained three grades. Pixel-wise spectra were extracted and preprocessed by wavelet transform and standard normal variate, and object-wise spectra (sample average spectra) were calculated. Principal component analysis (PCA) and independent component analysis (ICA) of object-wise spectra and pixel-wise spectra were conducted to select effective wavelengths. Pixel-wise PCA scores images indicated differences between two varieties and among different grades. SVM (Support Vector Machine), k-NN (k-nearest Neighbors Algorithm), and RBFNN (Radial Basis Function Neural Network) models were built to discriminate two varieties of raisins. Results indicated that both SVM and RBFNN models based on object-wise spectra using optimal wavelengths selected by PCA could be used for raisin variety identification. The visualization maps verified the effectiveness of using hyperspectral imaging to identify raisin varieties.

## 1. Introduction

Raisins are generally consumed as snacks, and they are also served as popular ingredients in many other food menus. Raisins are dried grapes which are rich in dietary fiber, carbohydrates with a low glycemic index, and minerals like copper and iron, with a low fat content [1,2]. In addition to their nutritional value, they also have medical value, such as regulating blood pressure for individuals with mild increases in blood pressure [2,3,4]. In general, raisins are important commercial products for the grape industry.

The commercial value of raisins differs according to the production area. In China, Xinjiang Uygur Autonomous Region is one of the major producing regions of grape, the perfect producing conditions and climates make it quite suitable for grape planting and deep processing. Variety is another important factor which influences the taste and nutritional compositions of raisins. To satisfy the demands of producers and consumers, different varieties of grapes are developed. Variety is one of the important factors in pricing the raisins. Varieties of raisins can be identified by specialist, experienced famers, and laboratory-based chemical analysis methods. To improve the identification efficiency, advanced non-destructive methods have been introduced, among which computer vision, spectroscopy, and spectral imaging techniques have shown great efficiency and potential for large scale detection at industrial level. Ma et al. achieved rapid non-destructive identification of apple varieties with 96.67% accuracy based on hyperspectral imaging [5]. Zhang et al. identified coffee variety using mid-infrared transmittance spectroscopy combined with pattern recognition algorithm [6]. Yang et al. developed a model for maize seed variety identification based on hyperspectral imaging [7].

Hyperspectral imaging is a technique combining computer vision and spectroscopy. Images of the study objects can be acquired for image analysis, and spectral information can be extracted from each pixel within the image for spectral analysis. A combination of image analysis and spectral analysis can also be explored. Hyperspectral imaging has been widely used in food analysis [8,9], and it has showed great potential in the grape industry. Fernandes et al. estimated grape anthocyanin concentration using hyperspectral imaging data. The squared correlation coefficient value was 0.65 compared to the values measured using conventional laboratory techniques [10]. Rodríguez-Pulido et al. found it was possible to assess the maturation stage in grape seeds based on the near-infrared spectra with prediction models and multivariate analysis methods [11]. Zhao et al. used hyperspectral imaging to identify different varieties of grape seeds. The results indicated that the variety of each single grape seed was accurately identified with 94.3% accuracy of the calibration set and 88.7% accuracy of the prediction set [12].

The general application of hyperspectral images is to conduct data analysis on a predefined region of interest (ROI) [12,13,14]. Spectral information is most widely used in hyperspectral image analysis, due to the advantage that spectral information can be precisely extracted from each pixel within ROIs. In general, pixel-wise spectra are averaged to build calibration models, and some researchers have focused on using pixel-wise spectra to build calibration models [15,16,17,18]. In fact, the size and the shape of raisins, which are key factors for the classification of different varieties, also play an important role in raisin grading within one variety. The raisin size can be influenced by the harvesting procedure of fresh grapes and the air-drying procedure, which can also beget irregular shapes of raisins in addition to storage.

The objective of this study is to explore the feasibility of using near-infrared hyperspectral imaging to identify raisin varieties. The specific objectives are: (1) exploring the influence of fruit size and shape in classification accuracy; (2) exploring spectral preprocessing of standard normal variate (SNV) in classification accuracy; (3) comparing performances of objective-wise analysis and pixel-wise analysis of SVM (Support Vector Machine), k-NN (k-nearest Neighbors Algorithm), and RBFNN (Radial Basis Function Neural Network) models.

## 2. Results and Discussion

### 2.1. Spectral Profiles

**Figure 1:**
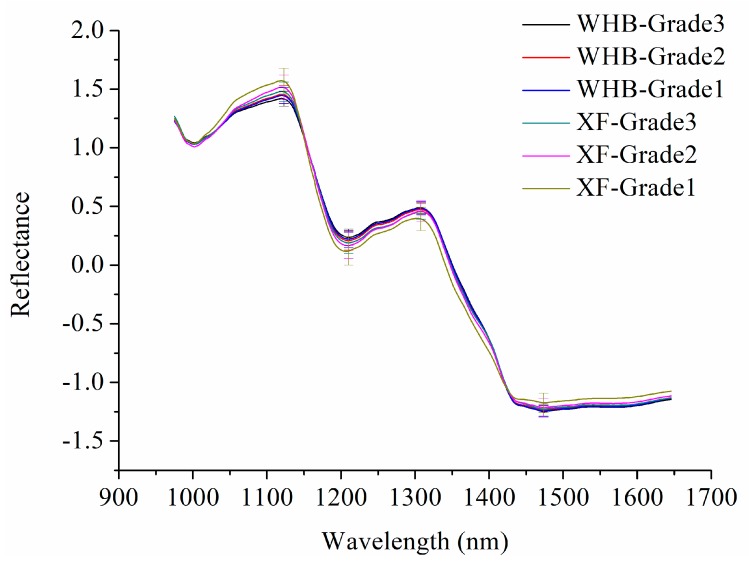
Average spectra with standard deviation (SD) of Wuhebai (WHB) and Xiangfei (XF).

In this research, 200 wavelength variables ranging from 975 to 1646 nm of hyperspectral images were studied. Figure 1 presents average spectra of each grade of raisins of Wuhebai (WHB) and Xiangfei (XF) with standard deviation (SD) at peaks and valleys (1123, 1210, 1308, and 1473 nm). The absorbance bands at 1123, 1210, and 1308 nm are largely attributed to the C–H stretching mode and overtone [19]. The wavelength around 1473 nm is a characteristic water wavelength [20]. It was obvious that a large proportion of overlap exists among eight curves, so it was necessary to conduct further study to make a better distinction between the two varieties of raisins.

### 2.2. PCA Scores Image Visualization

**Figure 2:**
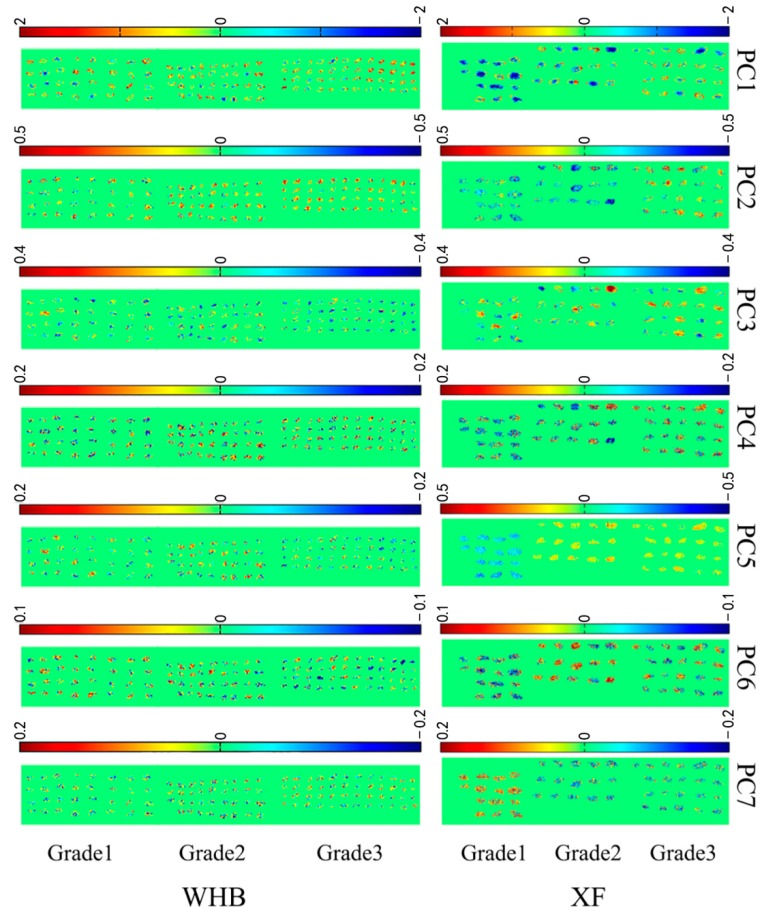
Scores image for the first seven principal components.

Pixel-wise PCA scores could be used to depict the PCA scores image. The first seven PCs explained over 99% of the total variance. Figure 2 shows visualized hyperspectral images of the first seven principal components (PC1–PC7) of two varieties of raisins. As can be seen from Figure 2, the warm color (yellow-red) accounted for the majority in WHB scores image of PC1 and PC2. In contrast to WHB scores image, the cold color (green-blue) was more obvious in XF scores image of PC1 and PC2, which revealed differences between two varieties. PCA scores image of PC5, PC6 and PC7 of XF exhibited obvious difference in color for Grade1 and two other grades, which showed differences among different grades. Although the PCA scores image could be used to distinguish different varieties and grades of raisins to some extent, it was necessary to conduct further study in order to obtain satisfactory classification results.

### 2.3. Effective Wavelength Selection

**Figure 3:**
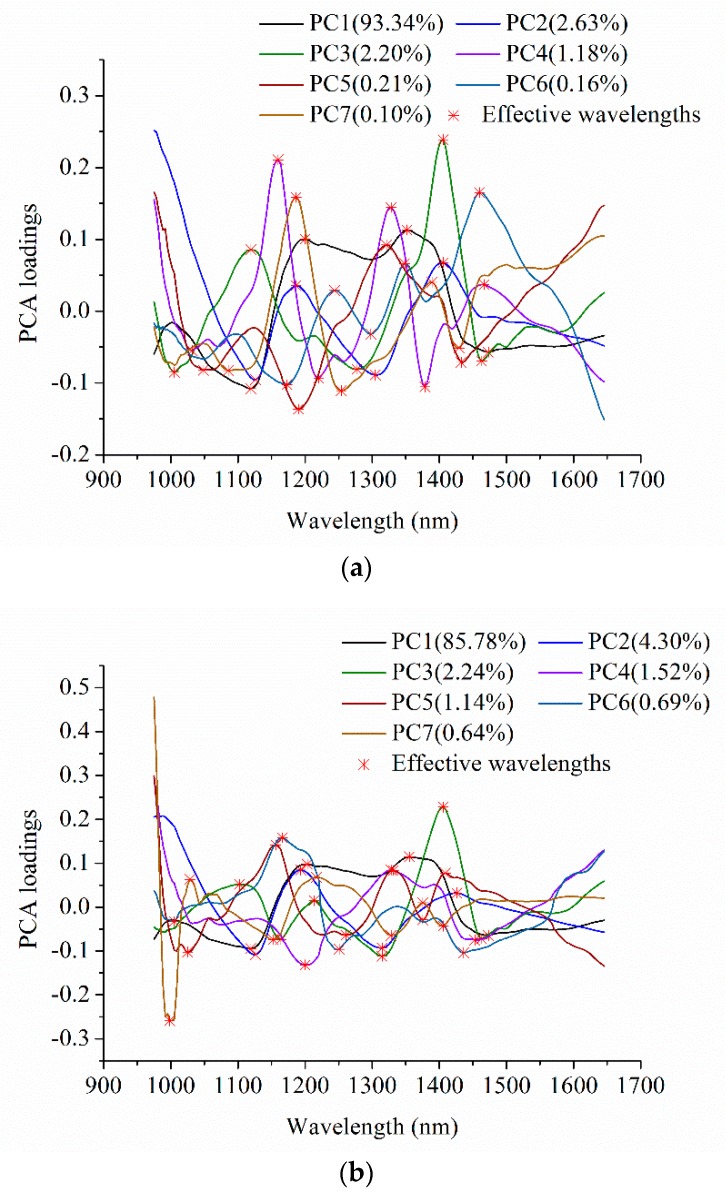
Corresponding optimal wavelengths selected by principal component analysis (PCA): (**a**) Object-wise analysis. (**b**) Pixel-wise analysis.

PCA loadings were used to select effective wavelengths for raisin cultivars classification. Since the first seven PCs explained over 99% of total variance, the loadings of these PCs were used. To examine the differences of object-wise analysis (average spectra) and pixel-wise analysis (pixel-wise spectra), PCA was conducted on both object-wise spectra and pixel-wise spectra of two different varieties of raisins. Figure 3 and Table 1 shows 20 or 17 optimal wavelengths selected by PCA based on object-wise analysis or pixel-wise analysis, respectively. PCA loadings plots of object-wise spectra and pixel-wise spectra were quite similar. As shown in Table 1, corresponding optimal wavelengths for object-wise spectra and pixel-wise spectra were nearly the same, with slight differences caused by different varieties and grades.

ICA was also conducted on object-wise spectra and pixel-wise spectra. To compare with PCA, the same numbers of optimal wavelengths are showed in Table 2.

### 2.4. Raisin Variety Classification Models Based on Different Grades

The PCA analysis above indicated that there were differences between different varieties of raisins, and there were also differences among different grades of raisins. Thus, grade was an important factor which influenced classification results of two varieties of raisins.

To evaluate the influence of different grades on model performances, samples from the same grade of raisins were formed as calibration set, and the remaining samples were used as prediction set. SVM (Support Vector Machine) models were built using effective wavelengths selected by PCA, and the results are shown in Table 3.

For the calibration model built based on Grade1, classification results of the calibration set were good for both varieties and prediction results of three grades of WHB were good, while prediction results of XF were poor. There were no correctly classified samples for Grade3 of XF. The SVM model built based on Grade2 exhibited better performance compared with SVM model based on Grade1. For the calibration model built based on Grade2, both the calibration set and prediction set obtained satisfactory results, and WHB and XF both obtained good classification results. When the calibration set was built according to Grade3, classification results of calibration set were good. The prediction results of the three grades of WHB were good, and the prediction results of Grade2 and Grade3 of XF were also good. However, the prediction result of Grade1 was poor with classification accuracy lower than 20%.

**Figure 4:**
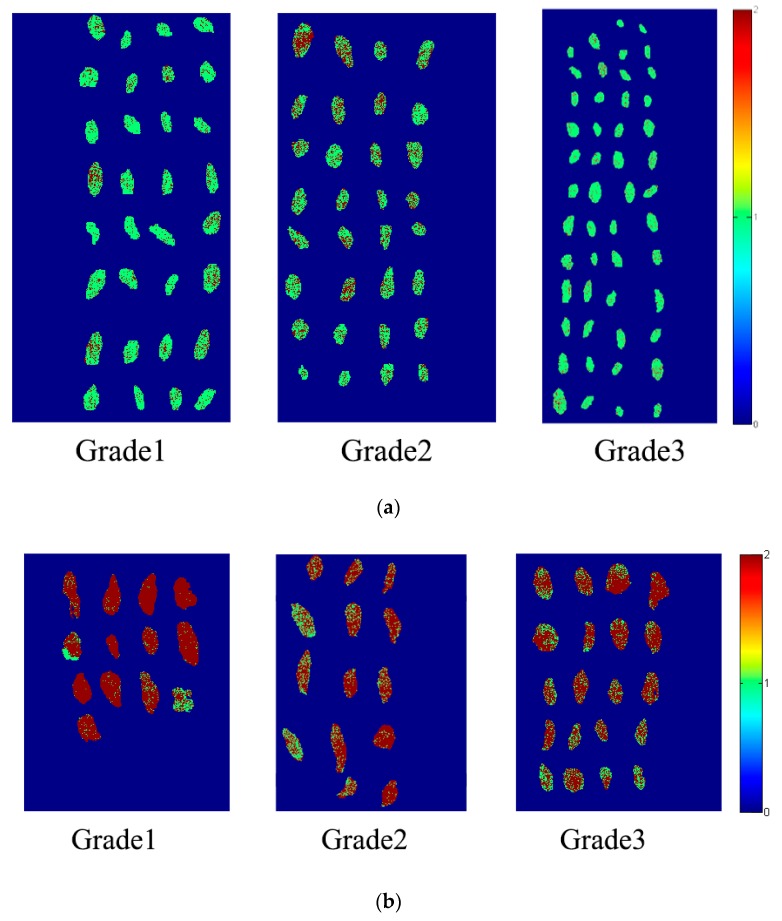
Classification results using pixel-wise spectra: (**a**) WHB; (**b**) XF.

These results revealed that different grades of raisins had influences on variety classification. As shown in Table 3, classification models based on Grade1 and Grade3 obtained poorer classification results compared with classification models based on Grade2. The reason might be that feature differences between Grade1 and Grade3 of XF were significant (for example the difference of sample size of different grades for same raisin variety was obvious as shown in Figure 4). The calibration set using Grade1 or Grade3 might not cover enough sample features used for PCA scores analysis.

Table 4 shows the results of SVM models built based on optimal wavelengths selected by ICA. Compared with Table 3, the calibration and prediction accuracies of SVM models based on Grade1 and Grade3 using optimal wavelengths selected by ICA were close to SVM models using optimal wavelengths selected by PCA. The accuracies based on Grade2 using optimal wavelengths selected by ICA were lower than SVM models based on PCA optimal wavelengths selection for both varieties of raisins.

### 2.5. Classification Results of Pixel-Wise and Object-Wise Spectra

According to Zhang et al. (2018) [21], pixel-wise spectra can be used to build classification models, and can achieve good prediction results on sample average spectra. The results of average spectra showed that sample size might be a factor influencing classification results. The advantage of hyperspectral imaging was to obtain spectral information of each pixel within the sample. Previous results have showed that pixel-wise spectra analysis has great value in hyperspectral image analysis [22,23]. For each variety of raisins, pixel-wise spectra were extracted. In all, there were about 300,000 pixels of each grade within the calibration sets of WHB and XF raisins. Establishing calibration models using such a great number of pixels (over 1,800,000 pixels) requires quite heavy computation. Selecting effective wavelengths could reduce the data volume significantly, but the data volume was still large. As for about 300,000 pixels of each grade of raisins, there might be redundant pixels for modelling, so representative pixels should be selected to reduce the data amount.

To select representative pixel-wise spectra, a calibration set selection procedure was proposed by Kang et al. (2004) [24]. Firstly, for pixel-wise spectra of all grades of a variety, the collected pixel-wise spectra were clustered into 3000 groups using the k-means algorithm. Secondly, the Euclidean distance between sample and group centroid was calculated, and the sample with smallest Euclidean distance was selected into the new calibration set.

SVM, k-NN, and RBFNN models were built using selected pixel-wise spectra or object-wise spectra, and prediction set was also formed based on selected pixel-wise spectra or object-wise spectra. The results are shown in Table 5. The value of sensitivity means the classification accuracy of WHB, and the value of sensitivity means the classification accuracy of XF. 

From Table 5, the results of SVM and RBFNN models using pixel-wise spectra to predict pixel-wise spectra were acceptable, with classification accuracy of calibration and prediction about 80%–90%. Meanwhile, the results of SVM and RBFNN models using pixel-wise spectra to predict object-wise spectra also obtained good results for calibration set, with 93.62% and 88.40% accuracy, respectively. Compared with SVM and RBFNN models based on pixel-wise spectral, the results of k-NN were slightly poorer, with accuracies varied from 40%–90%. Three models based on object-wise spectra all obtained acceptable results for the calibration set, with accuracies ranging from 87%–99%. SVM, k-NN and RBFNN models using object-wise spectra to predict object-wise spectra obtained better results for the prediction set compared with models using pixel-wise spectra to predict object-wise spectra, with all accuracies above 80%.

The overall results indicated that SVM and RBFNN models using object-wise spectra to predict object-wise spectra could be used to identify raisin varieties. Selection of representative samples was of significance for stable and accurate models, which should be further studied.

Table 6 shows the classification results for SVM, k-NN, and RBFNN models based on optimal wavelengths selected by ICA. The accuracies based on models using pixel-wise spectra to predict pixel-wise spectra or object-wise spectra were much lower than the same models using optimal wavelengths selected by PCA. The prediction set results of models using object-wise spectra to predict pixel-wise spectra were poor, with accuracies varying from 48%–63%. Models using object-wise spectra to predict object-wise spectra obtained acceptable results, with all accuracies above 90%. However, the calibration results of three models using object-wise spectra as the calibration set using optimal wavelengths selected by ICA were slightly lower than the results of three models using optimal wavelengths selected by PCA.

### 2.6. Prediction Maps of Raisin Variety Detection

Based on the developed models, prediction maps of different raisins varieties could be formed. According to the results in Table 5, we used the pixel-wise SVM model to form prediction maps. Raisin grades of the corresponding pixel were predicted to form classification maps, and prediction maps are shown in Figure 4. Pixel-wise prediction maps indicate that most of the pixels could be correctly classified. The prediction maps show clear a difference between WHB and XF according to different visualized prediction color.

## 3. Materials and Methods

### 3.1. Sample Preparation

**Figure 5:**
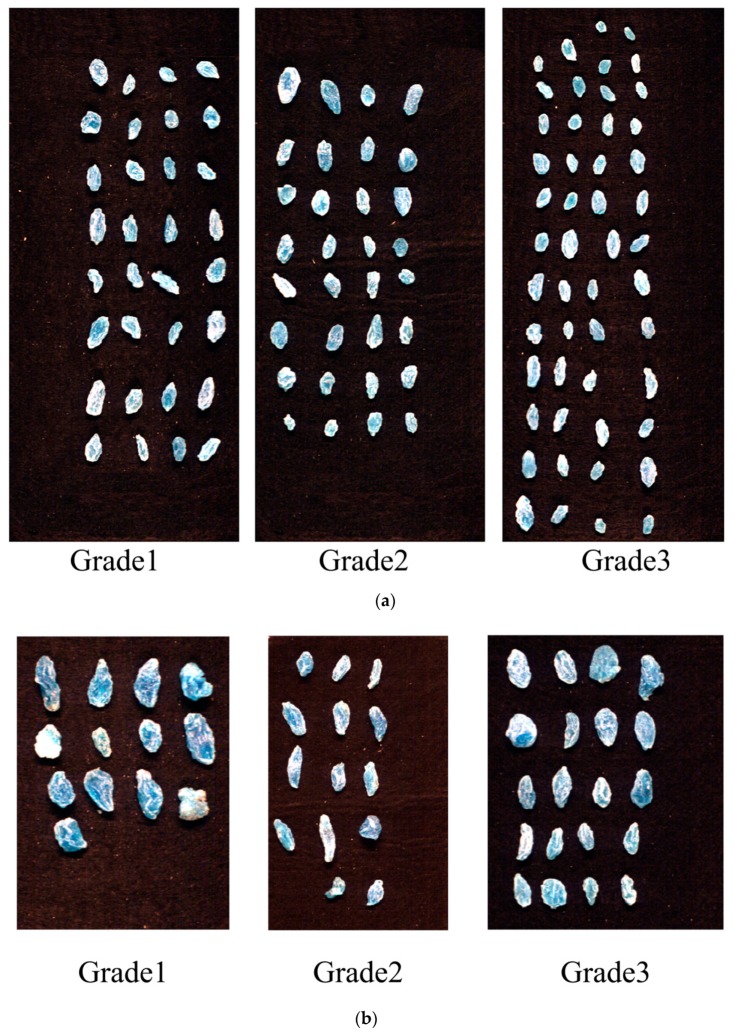
RGB images of the two varieties of raisins: (**a**) WHB; (**b**) XF.

Two varieties of raisins, including Wuhebai (WHB) and Xiangfei (XF), were collected from a local market in Shihezi, Xinjiang Uygur Autonomous Region, China. For each variety of raisin, three grades (Grade1-large size, Grade2-medium size, Grade3-small size) of raisins were manually collected according to the raisin size. For each grade, 450 g samples were divided into 30 groups (nearly 15 g per group) to acquire 30 hyperspectral images. Two varieties of raisins were all produced in 2017. RGB images of the two varieties of raisins are shown in Figure 5.

### 3.2. Hyperspectral Imaging System

**Figure 6:**
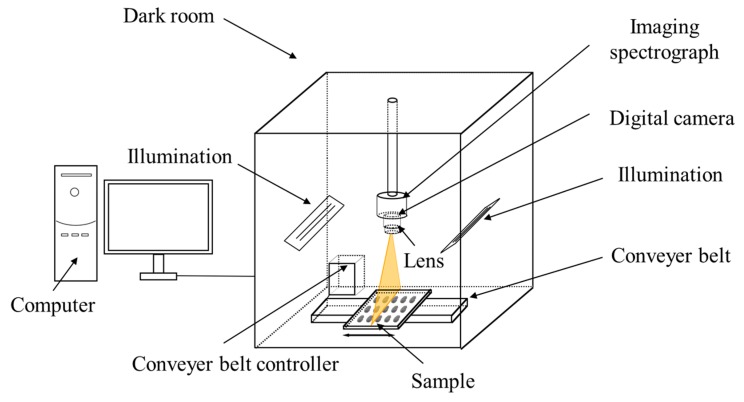
Hyperspectral imaging system.

The hyperspectral image acquisition was carried out using an assembled near-infrared hyperspectral imaging system with the spectral range of 975–1646 nm. The system consisted of an ImSpector N17E imaging spectrograph (Spectral Imaging Ltd., Oulu, Finland), a Xeva 992 camera (Xenics Infrared Solutions, Leuven, Belgium) installed with an OLES22 lens (Spectral Imaging Ltd., Oulu, Finland), two 150 W tungsten halogen lamps (3900 Lightsource, Illumination Technologies Inc., Elbridge, NY, USA) that were symmetrically placed and served as the light source, and a conveyer belt (Isuzu Optics Corp., Taiwan). The imaging system was controlled by the software (Xenics N17E, Isuzu Optics Corp., Taiwan), which can be used to calibrated and analyze the images as well. The sketch of the hyperspectral imaging system is presented in Figure 6.

### 3.3. Hyperspectral Image Acquisition and Correction

To acquire hyperspectral images, the distance between sample plane and the camera was set to 16 cm, the moving speed of the plate was set to 13.5 mm/s, and the exposure time of the camera was set to 4 ms. After adjustment, white reference image was collected by a white Teflon bar whose reflectivity is approximately 100%, and dark reference image was acquired by turning off the light source and covering the lens with lens cap whose reflectivity is about 0%. The white and dark reference images were used to calibrate the light intensity and reduce the dark current. For each group, one image was acquired. In all, 30 images were acquired for each grade of raisin.

After raw hyperspectral images acquisition, hyperspectral images were then corrected by the following equation:

(1) $$I_{c}=\frac{I_{r}-I_{d}}{I_{w}-I_{d}}$$

where *I_c_* is the corrected image, *I_r_* is the raw image, *I_w_* is the white reference image and *I_d_* is the dark reference image.

### 3.4. Spectral Data Preprocessing and Extraction

The hyperspectral image at 1119 nm was selected for background segmentation since the reflectance difference between sample and background was more obvious. To differentiate background from foreground, we set the segmentation threshold to 0.122 for hyperspectral image binarization.

After image correction, spectral information was extracted from hyperspectral images. Each raisin kernel was defined as the region of interest (ROI). Pixel-wise spectra within the ROI were firstly extracted, and wavelet transform (WT) was used for smoothing. Wavelet function of Daubechies 7 with a decomposition level of 3 was used to reduce random noises. After WT preprocessing, standard normal variate (SNV) was used to reduce the influence of scattering of pixel-wise spectra [25]. Then, average spectra calculated according to pixel-wise spectra within each ROI were used to represent the sample. In this study, pixel-wise spectra and average spectra were both used for analysis.

To extract spectral information, a binary image was obtained using the gray-scale image at 1199 nm, the background was set as 0 and the kernel regions were set as 1. The binary image was applied to the gray-scale images at each wavelength, and the background information was thus removed. After the background removal, pixel-wise spectra were extracted and preprocessed.

### 3.5. Sample Set Division

For each grade of raisin, 30 hyperspectral images were acquired. Hyperspectral images were randomly split into the calibration set and prediction set at the ratio of 2:1, with 20 hyperspectral images in the calibration set and 10 hyperspectral images in the prediction set for each grade.

### 3.6. Data Analysis Methods

#### 3.6.1. Principal Component Analysis

Principal component analysis (PCA) was used to explore the qualitative differences among different varieties of raisins [11,26,27,28]. In hyperspectral images, object-wise analysis and pixel-wise analysis were studied. For object-wise analysis, the average spectrum of each raisin kernel was used to conduct PCA; for pixel-wise analysis, pixel-wise spectra were used to conduct PCA. To explore the differences among raisins, the samples in the calibration set were used to conduct PCA. Then, scores values of each PC were then assigned to each kernel or each pixel to form the PCA scores image.

Hyperspectral imaging suffers from the large volume of data, and effectively reducing the data volume is of significance for data processing. There are also collinearity and redundancy in the spectra, which will affect the data analysis procedure. Variable selection is an effective strategy to reduce the data volume and select informative wavelengths. In this study, PCA loadings were used to select effective wavelengths. Loadings of each principal component (PC) indicate the correlation between the original variables and new feature variables. The higher the loading value is, the more important the variable is. The wavelengths with high absolute loading values can be selected as effective variables.

#### 3.6.2. Independent Component Analysis

Independent component analysis (ICA) is a technique which is widely used in feature selection and feature extraction. It extracts independent source signals which are statistical independent by linear or nonlinear transformation. Independent component (IC) is obtained by a high-order statistic. Given a spectral matrix *X*, *X* can be expressed as Equation (2):

(2) X=As

where *s* are the independent components (ICs) and *A* is the mixing matrix. For spectral data matrix *X*, *s* is unknown, and the general procedure is to find the estimation of s by the following equation:

(3) $$\overset{⌢}{s}=WX$$

where $\overset{⌢}{s}$ is the estimation of *s* and *W* is the weight matrix for unmixing.

The procedure to select optimal wavelengths is as follow [29]. The average absolute weight value of each variable in W is calculated, and the variables with larger average absolute weight values are selected as optimal wavelengths. To compare with PCA, the same number of optimal wavelengths was selected by ICA. Fast ICA proposed by Hyvarinen and Oja was used to perform ICA in this study [30].

#### 3.6.3. Discriminant Models

Support vector machine (SVM) is used to build models to classify different varieties of raisins. SVM is a supervised machine learning method, which is efficient to deal with linear and nonlinear data for classification and regression. For classification issues, SVM maps the original data into new feature spaces [31,32,33]. According to linearly separable data, a simple linear classifier can be constructed. For non-linearly separable data, the original data should be mapped into high-dimensional feature spaces so that the non-linearly separable issue can be transformed to a linearly separable issue. Kernel functions are the key for the mapping. Radial basis function (RBF) is a widely used kernel function with good performances for nonlinear data, and it was used as kernel function in this study. To conduct SVM with RBF kernel function, model penalty coefficient (C) and kernel parameter (γ) were determined by a grid-search procedure. In this study, the range of C and γ was 2^−8^–2^8^.

The k-nearest neighbors algorithm (k-NN) is a type of instance-based learning method used for classification and regression [34,35]. Both for classification and regression, a useful technique can be used to assign weight to the contributions of the neighbors, so that the nearest neighbors contribute more to the average than the more distant ones. The k-NN algorithm is among the simplest of all machine learning algorithms.

Radial basis function neural network (RBFNN) is an efficient feedforward neural network, which has the best approximation performance and global optimal characteristics that outperforms other feedforward networks, and has a simple structure as well as a fast training speed. On the other hand, it is also a neural network model that is widely used in pattern recognition, nonlinear function approximation, and other fields [36,37].

#### 3.6.4. Software and Model Evaluation

The performance of classification models was evaluated by the classification accuracy, specificity, and sensitivity [38]. Hyperspectral images analysis, spectral data extraction, spectral preprocessing, PCA analysis, SVM, K-NN, and RBFNN were conducted on Matlab R2014b (The MathWorks, Natick, MA, USA).

#### 3.6.5. Visualization of Prediction Maps

One of the advantages of hyperspectral imaging is that prediction maps can be formed to visualize the distribution of physical and chemical features. Object-wise or pixel-wise calibration models using spectra extracted from the hyperspectral images can be used to predict object-wise or pixel-wise features, and prediction maps can be formed with the predicted values [6,13,39].

## 4. Conclusions

Hyperspectral imaging was successfully used to identify different varieties of raisins. Three grades of raisins of Wuhebai and Xiangfei were studied. Object-wise and pixel-wise spectra were extracted. PCA analysis was firstly conducted to form PCA scores images, and scores images of the first seven PCs indicated the differences between different varieties and among different grades. PCA and ICA of object-wise spectra and pixel-wise spectra were conducted to select effective wavelengths. The overall results indicates that SVM models and RBFNN models using object-spectra to predict object-spectra based on optimal wavelengths selected by PCA both obtained acceptable results. The overall results showed that hyperspectral imaging was an effective technique to identify raisin varieties, and that both pixel-wise and object-wise could be used to build classification models. Selection of representative samples was important for building a stable and accurate model, and how to select representative samples should be studied in the future.

## Figures and Tables

**Table 1:** Corresponding optimal wavelengths selected by PCA.

Type of Analysis	No.	Optimal Wavelengths (nm)
Object-wise	20	1005, 1032, 1049, 1086, 1119, 1160, 1173, 1187, 1200, 1220, 1244, 1254, 1278, 1305, 1328, 1352, 1379, 1406, 1433, 1473
Pixel-wise	17	1005, 1029, 1103, 1119, 1164, 1200, 1214, 1251, 1261, 1315, 1328, 1355, 1375, 1406, 1426, 1436, 1473

**Table 2:** Corresponding optimal wavelengths selected by independent component analysis (ICA).

Type of Analysis	No.	Optimal Wavelengths (nm)
Object-wise	20	982, 985, 995, 999, 1002, 1009, 1012, 1015, 1019, 1022, 1025, 1029, 1032, 1035, 1039, 1042, 1046, 1049, 1052, 1056
Pixel-wise	17	1139, 1143, 1146, 1150, 1153, 1156, 1207, 1210, 1230, 1521, 1527, 1531, 1548, 1554, 1561, 1575, 1582

**Table 3:** Classification models based on different grade using optimal wavelengths selected by PCA.

WHB	XF	C ^4^	γ ^4^	Cal. Result		Pre. Results		
				WHB	XF	Pre. set	WHB	XF
Grade1 ^1^	Grade1	1	3.0	665/665	245/246	Grade3	1382/1382	0/602
						Grade2	930/931	22/453
						Grade1	380/380	99/116
Grade2 ^2^	Grade2	256	16	622/622	304/305	Grade3	1371/1382	559/602
						Grade2	305/309	146/148
						Grade1	1040/1045	323/362
Grade3 ^3^	Grade3	48.5	9.1	950/950	405/405	Grade3	419/432	197/197
						Grade2	658/931	434/453
						Grade1	1033/1045	51/362

**^1^** Grade1 represents large size; **^2^** Grade2 represents medium size; **^3^** Grade3 represents small size; **^4^** C and γ are parameters of SVM model.

**Table 4:** Classification models based on different grade using optimal wavelengths selected by ICA.

WHB	XF	C	γ	Cal. Result		Pre. Results		
				WHB	XF	Pre. set	WHB	XF
Grade1	Grade1	147.0	0.3	664/665	242/246	Grade3	1380/1382	0/602
						Grade2	931/931	17/453
						Grade1	379/380	100/116
Grade2	Grade2	147.0	48.5	606/622	255/305	Grade3	1360/1382	267/602
						Grade2	296/309	119/148
						Grade1	1014/1045	306/362
Grade3	Grade3	84.4	3.0	944/950	385/405	Grade3	409/432	197/197
						Grade2	487/931	393/453
						Grade1	899/1045	15/362

**Table 5:** Classification results for SVM, k-NN, and RBFNN models based on optimal wavelengths selected by PCA.

	Model	Parameter ^5^	Calibration Set			Prediction Set		
			Acc. ^6^ (%)	Sen. ^7^	Spe. ^8^	Acc. (%)	Sen.	Spe.
Pixel to pixel ^1^	SVM	(256, 5.28)	91.83	0.898	0.939	80.10	0.800	0.802
	k-NN	3	78.48	0.700	0.870	78.18	0.642	0.895
	RBFNN	7	88.40	0.842	0.926	80.89	0.797	0.819
Pixel to object ^2^	SVM	(256, 5.28)	91.83	0.898	0.939	93.62	0.785	0.998
	k-NN	3	78.48	0.700	0.870	83.82	0.464	0.992
	RBFNN	7	88.40	0.842	0.926	91.40	0.711	0.997
Object to pixel ^3^	SVM	(147, 9.12)	99.72	0.994	0.998	71.10	0.817	0.626
	k-NN	5	95.46	0.870	0.991	76.86	0.727	0.803
	RBFNN	3	99.78	0.994	0.999	54.14	0.819	0.317
Object to object ^4^	SVM	(147, 9.12)	99.72	0.994	0.998	99.12	0.987	0.993
	k-NN	5	95.46	0.870	0.991	94.06	0.839	0.982
	RBFNN	3	99.78	0.994	0.999	99.30	0.983	0.997

^1^ Pixel to pixel means to use models using pixel-wise spectra to predict pixel-wise spectra; ^2^ Pixel to object means models using pixel-wise spectra to predict object-wise spectra; ^3^ Object to pixel means to use models using object-wise spectra to predict pixel-wise spectra; ^4^ Object to object means to use models using object-wise spectra to predict object-wise spectra; ^5^ Parameters for SVM models are C and γ, parameter for k-NN is number of neighbors (k) and parameter for RBFNN is spread value; ^6^ Acc. means accuracy; ^7^ Sen. means sensitivity; ^8^ Spe. means specificity.

**Table 6:** Classification results for SVM, k-NN, and RBFNN models based on optimal wavelengths selected by ICA.

	Model	Parameter ^5^	Calibration Set			Prediction Set		
			Acc. ^6^ (%)	Sen. ^7^	Spe. ^8^	Acc. (%)	Sen.	Spe.
Pixel to pixel ^1^	SVM	(256, 16)	82.15	0.739	0.903	74.9	0.708	0.784
	k-NN	3	85.60	0.791	0.896	71.13	0.618	0.789
	RBFNN	6	78.92	0.695	0.884	76.74	0.797	0.819
Pixel to object ^2^	SVM	(256, 9.19)	82.15	0.739	0.903	78.63	0.271	0.998
	k-NN	3	85.60	0.791	0.896	79.58	0.393	0.962
	RBFNN	6	78.92	0.695	0.884	80.47	0.341	0.996
Object to pixel ^3^	SVM	(147, 84.45)	94.68	0.879	0.976	54.63	0.870	0.285
	k-NN	5	93.64	0.849	0.974	62.17	0.709	0.551
	RBFNN	3	93.96	0.851	0.977	48.34	0.565	0.417
Object to object ^4^	SVM	(147, 84.45)	94.68	0.879	0.976	93.81	0.863	0.969
	k-NN	5	93.64	0.849	0.974	90.58	0.805	0.947
	RBFNN	3	93.96	0.851	0.977	93.30	0.844	0.970

^1^ Pixel to pixel means to use models using pixel-wise spectra to predict pixel-wise spectra; ^2^ Pixel to object means models using pixel-wise spectra to predict object-wise spectra; ^3^ Object to pixel means to use models using object-wise spectra to predict pixel-wise spectra; ^4^ Object to object means to use models using object-wise spectra to predict object-wise spectra; ^5^ Parameters for SVM models are C and γ, parameter for k-NN is number of neighbors (k) and parameter for RBFNN is spread value; ^6^ Acc. means accuracy; ^7^ Sen. means sensitivity; ^8^ Spe. means specificity.
